# Distinct cholangiocarcinoma cell migration in 2D monolayer and 3D spheroid culture based on galectin-3 expression and localization

**DOI:** 10.3389/fonc.2022.999158

**Published:** 2023-01-12

**Authors:** Siriwat Sukphokkit, Pichamon Kiatwuthinon, Supeecha Kumkate, Tavan Janvilisri

**Affiliations:** ^1^ Department of Biochemistry, Faculty of Science, Mahidol University, Bangkok, Thailand; ^2^ Department of Biochemistry, Faculty of Science, Kasetsart University, Bangkok, Thailand; ^3^ Department of Biology, Faculty of Science, Mahidol University, Bangkok, Thailand

**Keywords:** 3D culture, spheroid, cholangiocarcinoma, galectin-3, cell migration

## Abstract

**Introduction:**

Cholangiocarcinoma (CCA) is difficult to cure due to its ineffective treatment and advanced stage diagnosis. Thoroughly mechanistic understandings of CCA pathogenesis crucially help improving the treatment success rates. Using three-dimensional (3D) cell culture platform offers several advantages over a traditional two-dimensional (2D) culture as it resembles more closely to *in vivo* tumor.

**Methods:**

Here, we aimed to establish the 3D CCA spheroids with lowly (KKU-100) and highly (KKU-213A) metastatic potentials to investigate the CCA migratory process and its EMT-associated galectin-3 in the 3D setting.

**Results and discussion:**

Firstly, the growth of lowly metastatic KKU-100 cells was slower than highly metastatic KKU-213A cells in both 2D and 3D systems. Hollow formation was observed exclusively inside the KKU-213A spheroids, not in KKU-100. Additionally, the migration activity of KKU-213A cells was higher than that of KKU-100 cells in both 2D and 3D systems. Besides, altered expression of galectin-3 were observed across all CCA culture conditions with substantial relocalization from inside the 2D cells to the border of spheroids in the 3D system. Notably, the CCA migration was inversely proportional to the galectin-3 expression in the 3D culture, but not in the 2D setting. This suggests the contribution of culture platforms to the alternation of the CCA cell migration process.

**Conclusions:**

Thus, our data revealed that 3D culture of CCA cells was phenotypically distinct from 2D culture and pointed to the superiority of using the 3D culture model for examining the CCA cellular mechanisms, providing knowledges that are better correlated with CCA phenotypes *in vivo*.

## 1 Introduction

Cholangiocarcinoma (CCA) is an epithelial malignancy of the biliary ducts. The global incidence of CCA is on the rising trend with the highest rate in Southeast Asia, especially in the northeastern part of Thailand (33.4 and 12.3 cases per 100,000 people in men and women, respectively) (1, 2). Studies of CCA patients in the regions with high parasitic prevalence revealed a strong association between chronic liver fluke and CCA carcinogenesis (1, 3, 4). CCA has also been implicated with biliary duct disorders, hepatitis B and C infection, and primary sclerosing cholangitis (5, 6). Asymptomatic and non-specific symptoms often make an early diagnosis of CCA difficult, causing patients to be diagnosed at an advanced stage (7). Surgical resection is usually represented as the standard therapy while radiation and chemotherapy are deemed less effective. However, the success rate of CCA treatment remains poor as the surgical treatment by liver resection and transplantation only yield ~25-40% 5-year survival rate (8). As a result, a profound understanding in CCA molecular pathways is crucially required to improve the treatment efficiency and to identify novel potent molecular target for future CCA therapy.

In order to thoroughly gain knowledge on CCA pathogenesis and carcinogenesis, *in vitro* studies using CCA cell lines have been implemented as tools to discern the cellular and molecular alteration of the disease without the involvement of patient participants or animal studies (9–11). Despite easy manipulation, the traditional *in vitro* 2D culture system lacks several biological cues that are established *in vivo*, posing major pitfalls in correlating the *in vitro* with *in vivo* results (12). Alternatively, an *in vitro* three-dimensional (3D) culture has therefore been developed to overcome these problems and bridges the gap between the 2D culture and the animal model. The 3D culture system has been established to be more resembling to *in vivo* tumor properties, including histomorphology, functions, and microenvironments in terms of cellular heterogeneity, nutrients and oxygen gradients, cell-cell interaction, matrix deposition, and gene expression (13–15). Currently, there are several methods to establish tumor spheroids including forced-floating method (16), hanging drop method (17), agitation-based approach (18), matrix- and scaffold-based culture (19, 20), and microfluid cell culture platforms (21, 22). Among these methods, the matrix-based cell culture recapitulates cellular organization and functions due to the presence of various types of cell-extracellular matrix (ECM) interactions (23). As a result, the ECM proteins are important for cell growth and differentiation as well as maintenance of cellular homoeostasis including the epithelial-mesenchymal transition (EMT). EMT is the central process which changes non-motile epithelial phenotypic cells into motile or invasive mesenchymal phenotypic cells (24). Its crucial role is evident toward cancer cell properties, especially its migration and metastatic potentials. Furthermore, EMT in hepatocellular and CCA has been found to involve with TGF-β signaling which leads to the inflammatory and fibrotic processes and pathogenesis (25).

Galectin-3, one key EMT proteins, is a β-galactoside-binding protein that is involved in many cellular functions of non-cancerous and cancerous tissues such as cell proliferation and survival, differentiation, inflammation, and cell migration and metastasis through the interaction between cells and ECM (26). Its structure consists of a carbohydrate recognition domain (CRD) and an N-terminal domain (ND), containing highly conserved proline and glycine-rich for 12 amino acid lengths. The ND can multimerize into dimers or pentamers when the ligand is present in CRD, transducing various extracellular and intracellular signals, causing diverse cellular activities (27, 28). Its expression is found in various organs, including the small intestine, kidney, colon, and lung tissue (29). Furthermore, galectin-3 is localized in diverse subcellular compartments, mainly in cytoplasm, cell surface membrane, and nucleus (30), responsible for the regulation of cell differentiation, apoptosis, and proliferation, respectively (29, 30). Remarkably, a previous study reported that galectin-3 could shuttle between nucleus and cytoplasm to mediate its functions (30). An aberrant expression of galectin-3 in distinct localization, specifically in cancers, has been associated with unique functions (9, 31, 32). In prostate cancer, galectin-3 is mainly localized at the cytoplasmic compartment of cancer specimens and its low expression is also related to poor progression of patients (31). Additional findings illustrated that cytoplasmic galectin-3 was associated with tumor progression (31, 33). Also, the loss of galectin-3 expression was associated with higher T-stage and the reduction of survival rate in renal carcinoma (32). A previous study on CCA showed that galectin-3 expression in intrahepatic CCA was lower than that in the normal bile duct (9). Moreover, gene suppression of galectin-3 in CCA cell lines also induces cell motility and cell invasion (9). In addition to cell mobility and invasion, galectin-3 displayed an association with chronic inflammation, enhancing the fibrotic pathogenesis in many types of tissues and cancers (34, 35). A recent study on intrahepatic CCA patients with COVID-19 infection displayed that the expression of galectin-3 was directly correlated with the level of MMP-9, another key indicator for the inflammation and the promotion of lung fibrosis (35). To date, there has been no report on other cell culture models, for example, 3D cultures and organoids, to confirm the role of galectin -3.

In this study, we established the 3D culture of CCA cells including lowly-metastatic KKU-100 and highly metastatic KKU-213A, formally known as KKU-M213, cells using the Matrigel-based culture to provide the cells with ECM environments (10). The cancer characteristics in terms of cell proliferation, cell migration, cell organization and protein expression between the 2D culture and 3D culture were evaluated. The level of proteins involved in cancer metastasis, including EMT and galectin-3, were compared among CCA cells and 2D- and 3D-based culture systems. Furthermore, the role of galectin-3 in cell migration of both culture systems was examined using gene silencing and rescue assays. Our results highlighted the distinct patterns of galectin-3 expression in both cells in 2D and 3D settings, resulting in different migration activities.

## 2 Materials and methods

### 2.1 Reagents

Ham’s F12 nutrient mix, Roswell Park Memorial Institute (RPMI) 1640, fetal bovine serum (FBS), penicillin/streptomycin, and Lab-Tek II 8-well chamber slide were purchased from Thermo Fisher Scientific (Waltham, MA, USA). Corning^®^ Matrigel^®^ Growth Factor Reduced (GFR) basement membrane matrix and cell recovery solution were acquired from Corning (Corning, NY, USA). Ribonuclease A and phenylmethylsulphonyl fluoride (PMSF) protease inhibitor were purchased from Sigma Chemicals (St. Louis, MO, USA). Bradford solution was purchased from Bio-Rad (Hercules, CA, USA). Hoechst 33342, TRITC-conjugated phalloidin, primary antibodies against galectin-3, ZEB-1, N-cadherin, E-cadherin, β-catenin, vimentin, horseradish peroxidase (HRP)-conjugated secondary antibodies against anti-rabbit and anti-mouse antibodies, Alexa Fluor^®^ 647-conjugated secondary antibody against anti-rabbit antibodies and SignalFire™ ECL Reagent were purchased from Cell Signaling Technologies (Denver, MA, USA). The primary antibody against β-actin was purchased from Sigma-Aldrich. Triton X-100 was obtained from Bio-Rad. Recombinant human galectin-3 (rGal-3) was purchased from Merck (Dorset, UK).

### 2.2 Cell lines and culture conditions

Human poorly differentiated KKU-100 and mixed papillary and non-papillary KKU-213A CCA cell lines, derived from Thai CCA patients, respectively, were employed to represent lowly and highly invasive CCA models in this study (10, 11). Both cell lines were purchased from the Japanese Collection of Research Bioresources Cell Bank and maintained in Ham’s F12 nutrient mix supplemented with 10% FBS, 100 U/ml penicillin and 100 μg/ml streptomycin. Human intrahepatic CCA RBE cell line, kindly gifted from Prof. David Bates (University of Nottingham, UK), was maintained in RPMI 1640 supplemented with 10% FBS, 100 U/ml penicillin and 100 μg/ml streptomycin. All cell lines were cultured at 37°C in a 5% CO_2_ incubator.

### 2.3 Tumor spheroid formation

To produce tumor spheroids of CCA cell lines, 35 µl of GFR Matrigel at a concentration of 5,000 µg/ml was filled into a 96-well plate and incubated at 37°C for 2 h. Then, 5 x 10^3^ CCA cells were seeded into Matrigel-coated wells and were further incubated for 15 min to allow cell attachment. Ham’s F12 nutrient mix supplemented with 10% FBS, 2% of 5 mg/ml Matrigel, 100 U/ml penicillin and 100 μg/ml streptomycin were added at a total volume of 120 µl each well. The 3D spheroids were formed and incubated 37°C in a 5% CO_2_ incubator and the culture medium were changed every two days.

### 2.4 Cell proliferation assay

For monolayer culture, ~5 x 10^3^ cells were seeded in a 96-well plate and incubated for 24 h at 37°C in 5% CO_2_ incubator. The cells were collected by trypsinization. The number of cells was counted by a dye exclusion method using 0.4% trypan blue. For 3D culture proliferation assay, the diameters of tumor spheroids were observed under a phase-contrast inverted microscope (Nikon model eclipse TS100, Minato, Tokyo, Japan) with 40x magnification for 5 fields per well and were measured using an ImageJ program (NIH image, National Institutes of Health, Bethesda, MD, USA). The cellular morphology was observed under a phase-contrast microscope using 100x magnification.

### 2.5 Migration assay for monolayer cells

Wound healing assay was performed by creating a scratch on the monolayer cells as our previous study (36). Migration activity of the cells was recorded at different timepoints. First, ~1 x 10^5^ CCA cells were seeded in a 24-well plate and incubated for 24 h. Then, culture medium was discarded, and the cells were washed with phosphate buffer saline (PBS). Scratches were created, and scattered cells were removed. Cells were then supplemented with Ham’s F12 nutrient mix containing 0.1% FBS, 100 U/ml penicillin and 100 μg/ml streptomycin. Wound images were captured under a phase-contrast microscope at 0 and 12 h to reduce the contribution of cell proliferation to fill the gap. The migration areas were determined using ImageJ analysis software version 1.8.0. The relative migration was calculated as the ratio between the difference in migration wound area at time 12 h relative to 0 h and migration wound area at time 0 h.

### 2.6 Migration assay for 3D CCA spheroids

The 3D migration assay for CCA spheroids was modified from a previously published protocol (37). Briefly, the 96-well plate was coated with 50 µl of 125 µg/ml GFR Matrigel and incubated at 37°C for 2 h. Twenty thousand (2 x 10^4^) CCA cells were then seeded on Matrigel-pre-coated wells and grew for 4 days. They were then collected and pipetted into Matrigel-coated wells. After that, the CCA spheroids were supplemented with 180 µl of Ham’s F12 nutrient mix, 0.1% FBS, 100 U/ml penicillin, and 100 μg/ml streptomycin. Spheroid images were taken at 0 and 12 h to prevent the effect of cell proliferation under a phase-contrast microscope. Next, cell migration areas were determined using ImageJ analysis software. The relative cell migration was subsequently calculated as the difference in the migrating area between time at 12h and 0 h.

### 2.7 Gene expression by qPCR

Total RNA was extracted from CCA cells in 2D and 3D culture systems using TRIzol reagent (Invitrogen, MA, USA). Then, 1 µg of the extracted RNA was used as a template to synthesize first strand cDNA using iScript™ cDNA synthesis kit (Biorad, CA, USA). Then, the relative gene expression of galectin-3 was evaluated using quantitative real-time polymerase chain reaction using specific galectin-3 primers and iTaq Universal SYBR Green Supermix (Bio-Rad, CA, USA). All reactions were performed in triplicate using the CFX Connect Real-Time system (Bio-Rad, CA, USA) with the following thermocycling conditions: initial denaturation at 95°C for 5 min, extension at 95°C for 30 s, 60°C for 30 s, 70°C for 60 s (40 cycles), and final extension at 70°C for 1 min. Actin gene expression was used as an internal housekeeping gene control to normalize gene expression between samples. The relative fold change was calculated using the 2^-ΔΔCq^ method. The gene primer sequences of galectin-3 F: 5’ GCCAACGAGCGGAAAATGG 3´, R: 5´ CAGGCCATCCTTGAGGGTTT 3´ and actin F: 5´ GCACAGACCTCGCCTT 3´, R: 5´ CTTGCACATGCCGAG 3´ were used.

### 2.8 Protein extraction and western blotting

Total proteins were retrieved using RIPA lysis buffer and a PMSF protein inhibitor. One hundred microliters of lysis cocktail were pipetted onto monolayer cells, which were harvested using a cell scraper. The supernatant was transferred into a microcentrifuge tube and was centrifuged at 4°C 15,000 x *g* for 10 min. The supernatant as protein lysate was collected for further analysis. For tumor spheroids, spheroids were collected using cell recovery solution and transferred into a 1.5 ml-microcentrifuge tube. The tumor spheroids were washed three times with cell recovery solution to eliminate ECM. The cell pellet was then filled with 50 µl of protein lysis cocktail and sonicated for 10 min on ice. The protein concentrations were determined using the Bradford assay. Then, 20 µg of protein sample was subjected to 10% SDS-PAGE at 100 V. The separated proteins were transferred to a nitrocellulose membrane using semi-dry electroblotting (Trans-Blot Turbo™ Transfer System, Bio-Rad laboratories, Hercules, CA, USA). The membrane was washed 3 times with tris buffer saline containing 0.1% tween and then incubated with 5% skim milk for 1 h to block non-specific binding. Primary antibodies for ZEB1 (1:1,000), E-cadherin (1:1,000), N-cadherin (1:1,000), β-catenin (1:500), vimentin (1:500), galectin-3 (1:500) and β-actin (1:10,000) were probed on the membranes at 4^°^C overnight. The membranes were then washed 3 times and incubated with HRP-conjugated secondary antibodies (1:500) for 1.5 h. The immunoreactivity of protein bands was determined using an enhanced chemiluminescent (ECL) substrate. Protein intensities were evaluated using ImageJ analysis software.

### 2.9 Immunofluorescence

For monolayer culture, cells were seeded into an 8-well Lab-Tek II chamber slide (Sigma-Aldrich, St. Louis, MO, USA) and incubated for 48 h. Then, cells were fixed using 4% paraformaldehyde for 15 min and washed with PBS for 5 min three times. The slide was blocked with 5% BSA for 30 min and then washed with PBS. Galectin-3 antibody (1:500) was added to the cells with incubation at 4°C overnight. The supernatant was discarded, and the cells were washed PBS for 5 min three times. Then, Alexa 647-conjugated secondary antibody was added to the cells with 1 h incubation in the dark. Hoechst 33342 (5 µg/µl) was added to the cells for 10 min in the dark. Cells were then washed with PBS for 5 min thrice and observed using a confocal laser scanning microscope (Olympus model FV10i-DOC, Shinjuku, Tokyo, Japan). For 3D culture, tumor spheroids were formed using a 3D Matrigel overlay culture for 6 days. Tumor spheroids were then retrieved with ice-cold PBS and centrifugation at 3,500 x *g* for 5 min. A tissue clearing protocol was adopted to improve image quality (38). Briefly, tumor spheroids were incubated with 25% formamide and 10% PEG for 10 min, followed by 50% formamide and 20% PEG for 1 h. Subsequently, the spheroids were washed extensively with PBS for 3 times and fixed with 4% paraformaldehyde for 1 h at room temperature. Tributyl phosphate solution was then added to the samples for 30 min at room temperature, followed by washing step with PBS 3 times. For cellular organization, the CCA spheroids were counterstained with TRITC-phalloidin and Hoechst33342 for actin filament and nucleus, respectively. For the localization of galactin-3, the CCA spheroids were incubated with galectin-3 antibody (1:500) overnight at 4°C and were washed with PBS 3 times. Alexa 647-conjugated secondary antibody was then added with 1 h incubation in the dark. Next, the stained CCA spheroids were wash with PBS. After that, Hoechst 33342 and TRITC-phalloidin were counterstained for 10 min in the dark. The CCA spheroids were then washed 3 times with PBS and transferred into an 8-well chamber slide before being observed using a confocal laser scanning microscope.

### 2.10 shRNA silencing of galectin-3

The galectin-3 shRNA plasmid (sc-155994-SH) containing a pool of three to five lentiviral vector plasmids each encoding galectin-3-specific 19-25 nucleotides (plus hairpin) shRNAs, and scramble shRNA plasmid-A (sc-108060) were purchased from Santa Cruz Biotechnology (Dallas, TX, USA). The knockdown experiment was slightly modified from our previous study (39). Briefly, galectin-3 shRNA or scramble plasmids were mixed with shRNA transfection reagent (sc-108061) and shRNA transfection media (sc-108062). Then, the plasmid mixture was transferred into the cells with 6 h incubation. Cells were then cultured in Ham’s F-12 nutrient mix supplemented with 20% FBS, 100 U/ml penicillin and 100 μg/ml streptomycin. After 48 h, transfected cells were selected using 1 µg/ml of puromycin in normal culture media. Non-transfected cells were removed, and transfected cells were maintained for verification using qPCR and Western blotting.

### 2.11 Galection-3 rescue experiment in galectin-3-knockdowned CCA cells

To investigate the effect of galectin-3 in CCA cell mobility in 2D culture, galectin-3-knockdowned CCA cells were pre-plated in 24-well plate and wound scratches were generated, followed by an incubation with 1.0 or 5.0 µg/mL rGal-3 containing medium (Ham’s F12 nutrient mix containing 0.1% FBS and 100U/ml penicillin/100 µg/ml streptomycin) for 6 or 12 h prior to experiment. For the 3D culture experiment, galectin-3-knockdowned CCA spheroids were pre-grown on Matrigel-coated wells for 4 days as previously described. Then, the galectin-3-knockdowned CCA spheroids were transferred to newly Matrigel-coated wells and incubated with 1.0 or 5.0 µg/mL rGal-3 containing medium (Ham’s F12 nutrient mix or RPMI 1640 containing 0.1% FBS and 100U/ml penicillin/100 µg/ml streptomycin) for 6 or 12 h prior to experiment. The culture medium containing 1% PBS was used as a control for the rescue experiments.

### 2.12 Galection-3 expression in CCA patients and survival analysis

To evaluate the correlation between galectin-3 expression and survival in CCA patients, publicly available web-based tools for analyzing RNA sequencing data (GEPIA), the Cancer Genome Atlas (TCGA) and the Genotype-Tissue Expression (GTEx) databases were used (40). The RNA sequencing data from 36 tumor specimens were divided into two groups according to their expression levels and overall survival and disease-free survival rates were created.

### 2.13 Statistical analysis

All experiments were performed at least in three biological replicates. All data were evaluated using two-tailed unpaired student’s T-test and presented as the mean ± standard error of the mean. The GraphPad Prism version 6.0 software was used for statistical analysis. Significant value cutoffs were set at *p* < 0.05.

## 3 Results

### 3.1 Characterization of CCA cells in 2D and 3D culture systems

**Figure 1:**
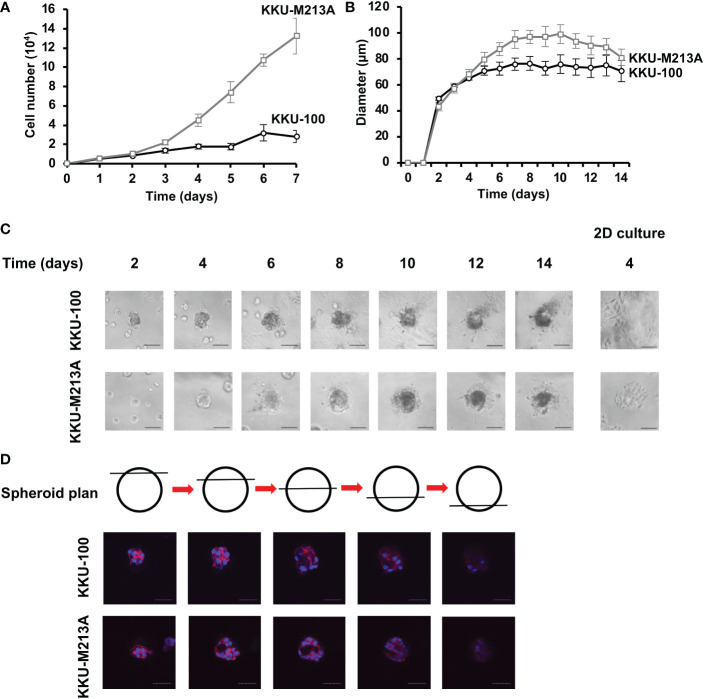
The growth rate of CCA cells in 2D and 3D cultures. **(A)** The growth rate of CCA cells in 2D culture as determined by cell counting. The cell numbers were counted every 24 h. Black circle and grey square represent KKU-100 and KKU-213A cells, respectively. **(B)** The growth rate of CCA cells in 3D culture as determined by diameter measurement. The diameter was measured every 24 h. Black circle and grey square represent KKU-100 and KKU-213A cells, respectively. The data represent means and ± standard error. **(C)** The tumor spheroid morphologies of KKU-100 and KKU-213A cells in 3D Matrigel-overlayed culture. The images were taken every 2 days using a phase-contrast microscope. The scale bars represent 100 µm. **(D)** Cellular organization of CCA spheroids. KKU-100 and KKU-213A cells were grown in 3D culture for 6 days. The CCA spheroids were permeabilized, blocked and subsequently stained with Hoechst 33342 for nucleus and TRITC-phalloidin for F-actin. Their structures were observed under a confocal microscope and Z-stack images were obtained. Red represents F-actin and blue represents nuclei. The scale bars represent 100 μm.

To determine the growth rate of both CCA cell lines in 2D and 3D culture systems, the numbers of cells and the diameters of CCA tumor spheroids were determined, respectively. For 2D monolayers, the numbers of both KKU-100 and KKU-213A cells gradually increased from 0 to 48 h. Then, a drastic growth of KKU-213A cells was observed from 72 h onwards, whereas the growth pattern of KKU-100 cells was much slower. The doubling time of KKU-100 and KKU-213A cells was 168 h and 54.6 h, respectively, confirming that highly metastatic KKU-213A exhibited a higher growth rate than lowly metastatic KKU-100 (**Figure 1A**). In 3D culture, the formation of both CCA spheroids were clearly observed at 48 h and the growth of both CCA spheroids was evaluated using the spheroid diameters (**Figure 1B**). During the first 4 days, KKU-100 and KKU-213A spheroids appeared comparable in sizes (**Figure 1B**). Then, the size of KKU213A spheroids significantly increased, exceeding that of KKU-100 spheroids. The largest sizes of KKU-100 and KKU- 213A spheroids were observed at the diameters of 76.4 and 99.2 µm on days 8 and 10, respectively. Interestingly, the size of KKU-213A spheroids reduced after day 10 of cultivation, while the size of KKU-100 spheroids was comparable after day 5 of cultivation (**Figure 1B**). Furthermore, the morphological structure of the growing CCA spheroids demonstrated a round colony outline of both CCA spheroids (**Figure 1C**). Additionally, the internal cellular organization of both CCA spheroids was examined using a confocal microscope. Z-stack images showed that both CCA spheroids possessed mass-forming properties with distorted cell rearrangement, oppositely observed in 2D CCA monolayers (**Figure 1D**). Notably, the highly metastatic KKU-213A developed luminal space inside its spheroids whereas lowly metastatic KKU-100 possessed a relatively uniformed spheroids, indicating a distinctively internal cellular organization in 3D culture.

### 3.2 Migration ability of CCA cells in 2D and 3D culture systems

**Figure 2:**
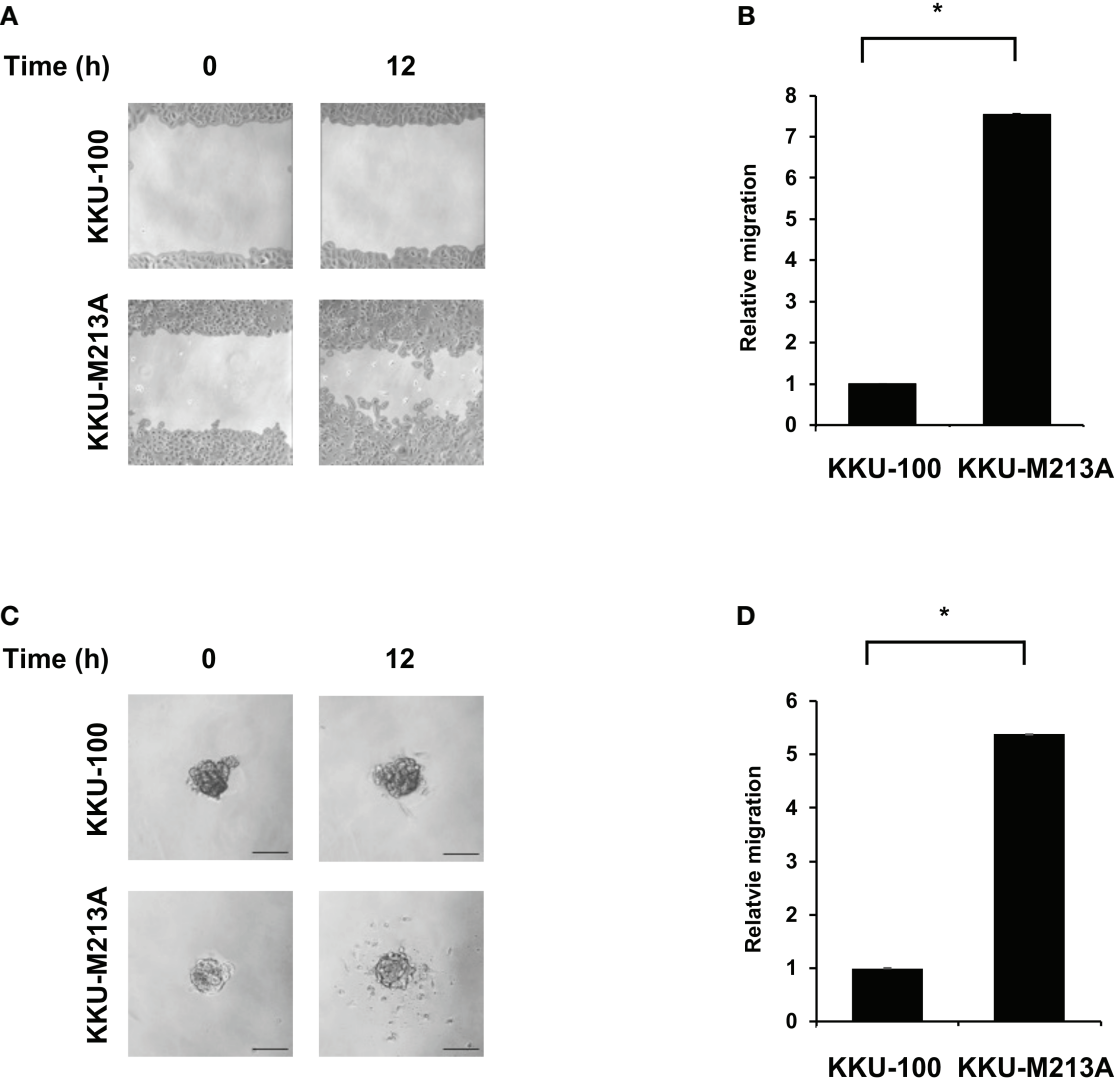
Migration activity of CCA cells in 2D and 3D cultures. KKU-100 and KKU-213A cells were grown as monolayers and spheroids for 4 days. **(A)** Wounds were created on CCA monolayer cells and size of the wound was photographed at 0 and 12 h following the wound scratch. **(B)** The relative migration is shown as the average value of migration index compared to KKU-100. **(C)** Representative images of 3D culture migration at 0 h and 12 h. The scale bars represent 100 μm. **(D)** The relative migration is represented by bar graph of the percentage migration area of KKU-213A in 3D culture compared with KKU-100. Data were presented as mean ± standard error, which were derived from three independent experiments, *p < 0.05.

The migration activity of CCA cells was observed using the wound healing assay for 2D culture and 3D migration assay for 3D culture. For the 2D wound healing assay, KKU-100 cells exhibited significantly lower migration, compared with KKU-213A cells (**Figures 2A**, **B**). Interestingly, KKU-100 barely migrated toward the scratch wound area, indicating the incompetence in cell mobility of KKU-100. Also, the 3D migration assay showed that KKU-100 spheroids substantially possessed lower migratory ability, compared to KKU- 213A spheroids which exhibited approximately ~5.1-fold migrative activity (**Figures 2C**, **D**). Although the migrative activities between 2D and 3D culture systems were not readily comparable, these findings demonstrated that KKU-213A cells exhibited higher migrative potential compared with KKU-100 cells in both 2D and 3D cultures.

### 3.3 Differential EMT protein expression in 2D and 3D CCA cultures

**Figure 3:**
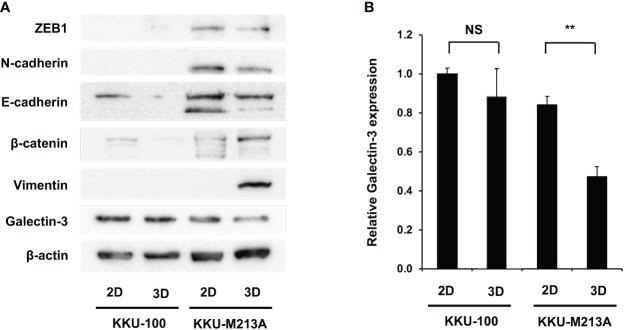
Migration-related expression in CCA cells in 2D and 3D culture systems. **(A)** CCA cells were grown as monolayers and spheroids for 6 days. Cells were harvested and lysed. Protein expression was determined by Western blotting. Migration-related proteins were compared between the two culture systems in each cell line. β-actin was used as an internal control. **(B)** Expressions of galectin-3 was quantified as relative intensity to β-actin. The data represent means and ± standard error. **p < 0.01, NS represents not significant.

**Figure 4:**
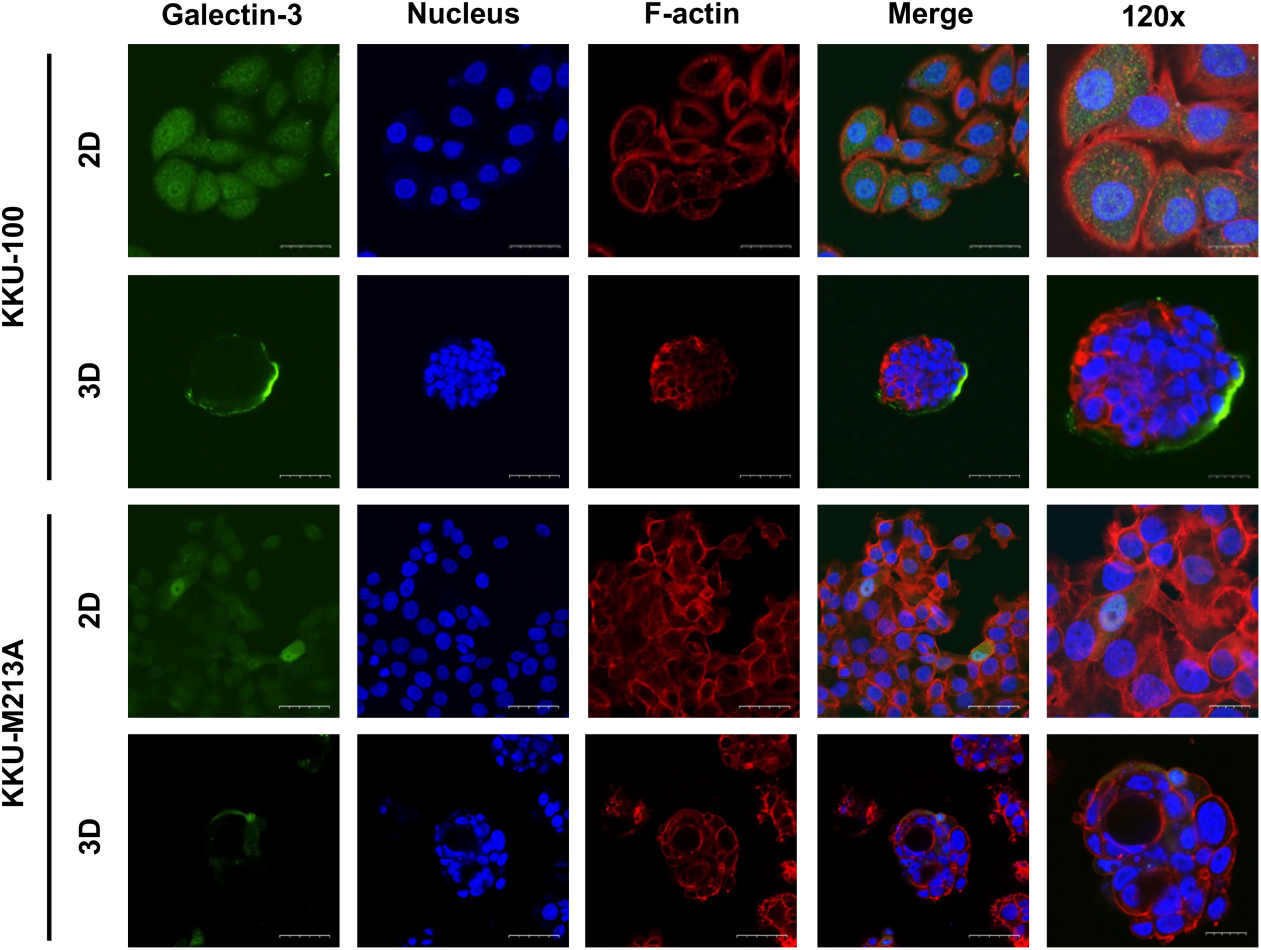
Localization of galectin-3 in 2D and 3D CCA cells. CCA cells were cultured as monolayer and spheroids for 10 days before harvesting for the immunofluorescence. CCA cells were fixed, permeabilized and blocked before staining with galectin-3 antibody (green). TRITC-phalloidin and Hoechst 33342 were used as counterstains for F-actin (red) and nuclei (blue), respectively. The tumor spheroid images were taken under a confocal microscope using 60x and 120x magnifications. The scale bars in 2D and 3D systems indicate 25 µm and 10 µm, respectively.

To further understand the role of EMT in 2D and 3D CCA migration, EMT markers including β-catenin, together with epithelial (E-cadherin), and mesenchymal markers (N-cadherin, vimentin, ZEB1) were investigated at a protein level in both CCA monolayers and spheroids (**Figures 3A**, **B**). For 2D monolayers, lowly metastatic KKU-100 and highly metastatic KKU-213A cells displayed no expression of vimentin. Moreover, the 2D KKU-100 cells demonstrated decreased expression of ZEB1, N-cadherin, and E-cadherin compared to the KKU-213A cells. Intriguingly, galectin-3, a multi-functional protein which has various ligands and locations, was expressed at a higher level in KKU-100 cells in relative to KKU-213A monolayers. In addition, the 3D KKU-100 spheroids exhibited reduced expression of ZEB1, N-cadherin, and E-cadherin while overexpressed galectin-3, compared to the 3D KKU-213A spheroids (**Figures 3A, B**). These results unveiled that galectin-3 was differentially expressed across culture conditions and CCA cell lines, causing it to be a good EMT candidate to examining the effect of EMT proteins on CCA migration. Subsequently, the intracellular localization of galectin-3 was examined to draw a correlation between cellular localization and migratory activities using immunofluorescence technique (**Figure 4**). In 2D culture, galectin-3 expressed throughout the nucleus and cytoplasmic compartment of the cells with a higher fluorescence intensity in KKU-100 than KKU-213A cells. In contrast, in 3D culture, galectin-3 was distinctively located on the outer layer of both KKU-100 and KKU-213A spheroids. Furthermore, KKU-100 spheroids had higher galectin-3 expression than KKU-213A spheroids, which is in accordance with Western blot analysis (**Figure 3**). Altogether, our data point to the different patterns of expression and localization of galectin-3 in 2D and 3D settings of highly and lowly metastatic CCA cells which potentially correlate with migratory activities in CCA.

### 3.4 Differential expression of galectin-3 revealed distinct migration activities in 2D and 3D CCA cells

**Figure 5:**
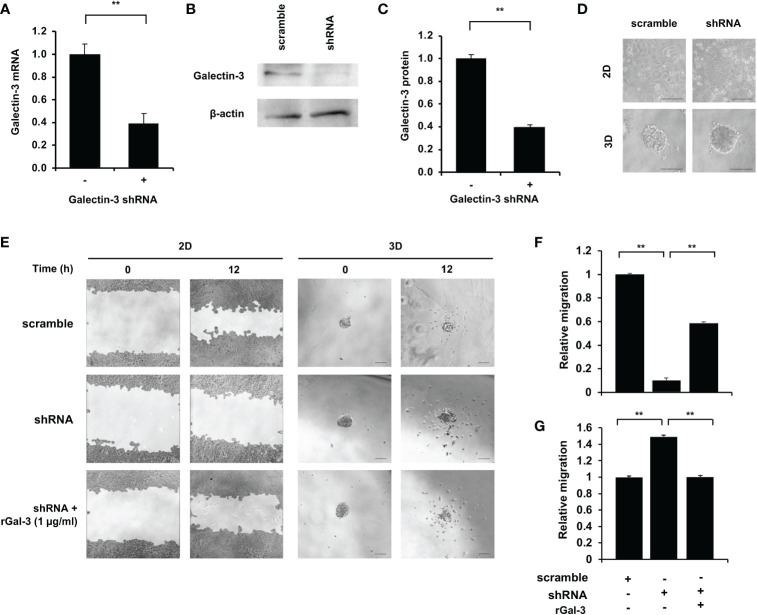
Effect of galectin-3 on 2D and 3D KKU-213A cell migration. **(A)** The relative galectin-3 mRNA level of KKU-213A knockdown cells compared to the control. **(B)** Representative galectin-3 immunoblots of KKU-213A cells subjected to *gal-3* and scrambled shRNA. β-actin was used as the loading control. **(C)** The relative galectin-3 protein expression of KKU-213A knockdown cells compared to the control. **(D)** The morphology of galectin-3 knockdown cells in 2D and 3D cultures. The 2D culture images were taken before cells reached confluence and 3D culture images were taken on day 6. **(E)** Control and galectin-3 knockdown KKU-213A cells were grown as monolayer or tumor spheroids for 4 days. rGal-3 was treated during the migration assay. The migration area of KKU-213A monolayer and tumor spheroids were collected at 0 h and 12 h. The scale bars represent 100 µm. **(F, G)** Bars represent the relative migration of galectin-3 knockdown cells with and without the treatment of rGal-3 in **(F)** 2D and **(G)** 3D conditions compared to the control cells. The data represent means and ± standard error. **p < 0.01.

Galectin-3 gene knockdown and recombinant galectin-3 (rGal-3) rescue assays were employed to investigate the correlation between galectin-3 and migration ability in CCA cells. KKU-213A was chosen as a CCA model due to the most distinguishable galectin-3 expression and migration ability. Gal-3-shRNA plasmids were transfected into the KKU-213A cells and showed a diminution in galectin-3 in both transcriptional and translational levels (**Figures 5A–C**). Additionally, the morphological study revealed no change in cellular structure in a comparison to its control cells in both 2D and 3D cultures (**Figure 5D**). We then performed the wound-healing assay for monolayer cells and the 3D migration assay for tumor spheroids, comparing between galectin-3-knockdown and control KKU-213A cells. The galectin-3-knockdown KKU-213A monolayers exhibited significantly lower cell migration compared to the control KKU-213A cells and their migration was resumed after adding the rGal-3 (**Figures 5E, F**). In contrast to 2D wound healing, the tumor spheroids of galectin-3-depleted KKU-213A cells revealed ~1.5-fold higher cell migration ability compared to KKU-213A spheroid control (*p* < 0.01) (**Figures 5E, G**). Furthermore, the rescue experiments displayed a strong adverse correlation between galectin-3 and migration ability in the 3D KKU-213A spheroids. To further confirm the role of galectin-3 on CCA cell migration, we additionally performed a stable knockdown of galectin-3 in RBE cells using shRNA transfection and evaluated cell migration in both 2D and 3D settings. The results coincided well with the findings observed for KKU-213A, suggesting the implication of galectin-3-associated pathways in 2D and 3D cultures of CCA cells (**Supplementary Figure 1**).

### 3.5 Clinical association between the expression of galectin-3 and overall survival in CCA TCGA database

**Figure 6:**
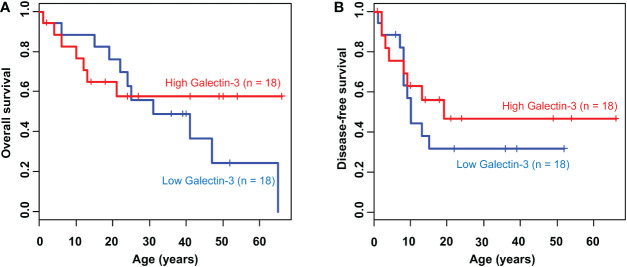
Kaplan-Meier survival curve of CCA patients according to galectin-3 expression. The cumulative **(A)** overall survival (*P* = 0.54) and **(B)** disease-free survival (*P* = 0.41) plot stratified by the level of galectin-3 expression.

As previously demonstrated, the expression and localization of galectin-3 in 3D CCA spheroids were explicitly deviated from that in 2D CCA monolayers and it was negatively correlated with the CCA migration ability. According to the high metastatic phenotype, poor prognosis and low overall survival were witnessed in a variety of cancers (26, 32). We then used Kaplan-Meier analysis to validate the correlation of galectin-3 and survival in CCA patients using clinical TCGA database (n =18). The results showed that CCA patients with low galectin-3 expression, albeit not statistically significant, seemed to exhibit poor prognosis (**Figure 6**). The propensity of these data coincided with our experimental results. Collectively, we propose that the 3D CCA culture, which potentially recapitulate an CCA *in vivo* condition, provided knowledge on the probable negative association between galectin-3 and cell migration, leading to clinically lower survival outcome.

## 4 Discussion

CCA is a cancer arising from the epithelial cells of biliary trees. The incidence of CCA in Thailand is extremely high, especially in the Northeast part of Thailand (2, 3). Treatment failure for CCA is common due to patient presentation at late stages, thereby often causing metastasis (1). Hence, further understanding in the cellular and molecular pathways of CCA is urgently required to enhance the treatment efficiency. The use of a conventional 2D *in vitro* system in CCA research could greatly fulfil the fundamental knowledge on cellular mechanisms of CCA (9, 36, 41, 42). Nevertheless, the 2D cell monolayer culture lack a number of vital biological signals and intracellular interactions such as cell-cell and cell-extracellular matrix interactions, normally observed *in vivo* (23). Recently, an *in vitro* 3D culture in which the cells are surrounded by ECM proteins, could potentially recapitulate *in vivo*-like environment and render *in vivo* characteristics, bridging and overcoming the experimental gap (23). Although, there are a few studies on CCA 3D culture, none have attempted to compare the characteristics of CCA migration in 2D and 3D culture. Hence, we established the 3D CCA culture model using Matrigel as a scaffold and evaluated the migration ability of lowly and highly metastatic CCA cells in 2D and 3D settings with a reference to the expression of galectin-3.

3D culture produces cell aggregates or tumor spheroids *in vitro*, which are more physiologically relevant to tumor tissues than the traditional 2D culture. While the cells in 2D culture are uniformly enriched with both oxygen and nutrient, tumor spheroids receive differential amounts of oxygen and nutrients, of which cells inside the core are exposed to less amount than the cells at the border, resulting in the necrotic core (43–45). At an early stage, the size of CCA tumor spheroids increased rapidly. Smaller spheroids thoroughly allow diffusion of oxygen and nutrients sufficient for their growth. When the size of tumor spheroids increases, cells tend to disassociate from spheroids, which could be resulted from the insufficient amount of oxygen and nutrients. This phenomenon can also be observed in other types of cancer. For example, the proliferation rate of endometrial cancer cells, including Ishikawa, RL-95-2, KLE, and EN-10780 reduced when grown in 3D reconstituted basement membranes (46). The growth rate of colorectal cancer cells, including CACO-2, DLD-1, HT-29, 3w480, LOVO, and COLO-206F, decreased when cultured on the laminin-rich-extracellular matrix compared to the 2D culture (47). The structures of tumor spheroids can differ even if they are from the same type of cancer. Here, we report mass-forming spheroids with disorganized structures in both KKU-100 and KKU-213A cells. Confocal images revealed the lumen formation in KKU-213A spheroids, not in KKU-100 spheroids. The luminal structure was also observed in other types of cancer such as breast and colorectal cancer spheroids (48, 49). The hollow formation was contributed to the TRAIL-mediated autophagy or caspase-induced apoptosis as a result of hypoxia and nutrient deprivation condition, typically observed in the 3D culture (48, 50, 51). Additionally, in the colorectal cancer, there was a strong relationship between hollow-forming spheroids and tumor metastasis and poor prognosis (49). Cancer stem cells in primary colorectal tumors could moreover be identified using the 3D culture method (51). Furthermore, the fluorescent signals from the confocal microscope were dimmed at the lower z-stack due to the limitation of laser at z-depth around 20–30 µm. This phenomenon has been noted in tumor spheroids produced from 25 breast cancer cell lines, which were categorized into 4 groups based on their organization and cell-cell interaction. These structure formations depend on the properties of each cell line, such as metastatic properties (52).

Our data showed that the KKU-213A compared to KKU-100 cells exhibited higher migration activity in both 2D and 3D settings. Our scratch wound healing results in 2D culture were comparable to the previous data using the Transwell migration assay (53). Additionally, KKU-213A cells exhibited both collective and individual cell migration while KKU-100 cells showed only collective migration. In 3D culture, the migration activity of CCA tumor spheroids was measured on Matrigel-coated wells to preserve adhesion molecules resembling the tumor microenvironment. Both CCA spheroids migrated individually rather than collectively migrated and some cells represented a mesenchymal phenotype, elongated cell shape, which is the malignant feature of cancer (54).

The migration of tumor cells is regulated by various pathways. The central pathway of migration is EMT, which is the process that allows non-motile epithelial cells to become motile mesenchymal cells. EMT is contributed by many proteins and transcription factors. To migrate, E-cadherin expression, which is an epithelial marker, has been reported to be reduced to suppress epithelial phenotypes (55). Then, β-catenin, which normally binds to the cytosolic part of E-cadherin is released and moves into the nucleus to turn on the target genes such as Snail1 and MMP-7 (56). The β-catenin accumulation in the nuclei is often found with the loss of E-cadherin expression (57). These data are correlated with our results showing that reduced E-cadherin and induced β-catenin expression in 3D CCA culture, reflecting the malignant form of CCA cells. Among the mesenchymal markers investigated, we found the reduction of ZEB1 and N-cadherin expression, which function as an EMT inducer and a mesenchymal marker, respectively. As galectin-3 interacts with and regulates other proteins, which contribute to EMT during tumor cell migration and invasion. We therefore further investigated the expression and localization of galectin-3 in KKU-100 and KKU-213A in both 2D and 3D culture. Our results revealed that galectin-3 expression was lower in highly metastatic KKU-213A compared with lowly metastatic KKU-100 cells and was significantly reduced in KKU-213A spheroids, but not in KKU-100. These results agreed with a previous study that reported the association of decreased expression of galectin-3 with the metastatic potential of liver fluke-associated cholangiocarcinoma (9). Although the vast majority of research focused on galectin-3 expression, a few studies have attempted to investigate its localization. In 2D culture, galectin-3 was uniformly distributed in KKU-100 cells at the higher level compared to KKU-213A cells. So, the evenly distributed galectin-3 in 2D CCA monolayers might confer additional functions on the 2D CCA cells, interfering with the migratory activity of CCA cells (26). In contrast, galectin-3 in 3D culture was strongly expressed at the border of KKU-100 spheroids, but to a much lesser extent in KKU-213A spheroids. Typically, the formation of 3D spheroids promotes the expression of the cell adhesion molecules on the cell surface membrane and, hence, increased cell-cell interaction which could recruit the accumulation of galectin-3 near the edge of spheroids (58). Noticeably, the intensely high membrane-associated galectin-3 in 3D epithelial KKU-100 spheroids might be attributed to the demand of the cells on interacting with Matrigel and galactoside-conjugated glycans on their adjacent cells, enhancing the cell attachment and reducing the cell migration activity (58). Besides, galectin-3 might interact with ECM and form a tumor capsule, a fibrotic capsule that harasses migratory cells. This tumor capsule has been shown as a mechanical barrier against local invasion (59).

The verification of the association between galectin-3 and CCA cell migration was performed in KKU-213A cells, due to its most distinguishable galectin-3 expression and migratory activity. The galectin-3 gene knockdown showed contradictory observations between 2D and 3D cell migration which was already discussed in the abovementioned paragraph together with galectin-3 localization and intensity. In addition, other cancer studies also reported contrast cell mobility between 2D and 3D cultures (33, 60, 61). Notably, it has been demonstrated that the suppression of galectin-3 *via* siRNA in 2D highly metastatic KKU-213B cells, formally recognized as KKU-M214, led to the promotion of cell migration (9). These could potentially be due to the cell line-specific behaviors which exclusively displayed distinct phenotypes including differentially gene expression profiles (10). Furthermore, although Kaplan-Meier analyses denoted non-significant correlation between galectin-3 overall and disease-free survival rates, which was similar to the work reported previously, however, lower galectin-3 expression has been significantly associated with lymphatic metastasis (9). Also a recent study hinted to the therapeutic potential of galectin-3 for treating intrahepatic CCA patients (35). Hence, our data revealed the differential expression level of galectin-3 in a relation to culture systems. Also, the diminished galectin-3 in the 3D spheroid culture system was inversely correlated with CCA cell migration. This 3D cell migration behavior potentially pointed to the closer resemblance of that in CCA *in vivo* conditions.

## 5 Conclusions

In summary, we established and characterized the 3D lowly- and highly-metastatic CCA spheroids. The 3D CCA spheroids demonstrated distinct patterns of growth and migration, compared to the 2D CCA monolayers. We further revealed differential expression and localization of galectin-3 and other EMT proteins in different culture settings. Galectin-3 uniquely accumulated at the outer surface of the 3D CCA spheroids, potentially facilitating the cell attachment and cellular binding characteristics. The expression of galectin-3 produced by the 3D CCA spheroids displayed a negative correlation with cell migration, which coincided well with previous observations in the clinical data. Altogether, the disparity in the culture systems described here illustrates the advantage of employing a 3D culture, which more resembles to *in vivo* conditions in investigating the CCA pathogenesis and carcinogenesis. It could potentially be developed as a CCA drug screening platform that offers a stronger correlation and more accurate interpretation to *in vivo* conditions.

## Data availability statement

The original contributions presented in the study are included in the article/**Supplementary Material**. Further inquiries can be directed to the corresponding author.

## Author contributions

SS and TJ designed and conceived the study. Material preparation, data collection and analyses were performed by SS, PK, SK. Analyses of the data were performed by SS, PK and TJ. The first draft of the manuscript was written by SS and PK and all authors commented on previous versions of the manuscript. All authors contributed to the article and approved the submitted version.
