# Supplementary material for: Distinct cholangiocarcinoma cell migration in 2D monolayer and 3D spheroid culture based on galectin-3 expression and localization
Source: Front Oncol. 2023 Jan 12;12:999158. doi: 10.3389/fonc.2022.999158 (PMC9881414; doi:10.3389/fonc.2022.999158)
Supplement: Supplementary file 1 [file Image_1.pdf]

Supplementary Figure 1

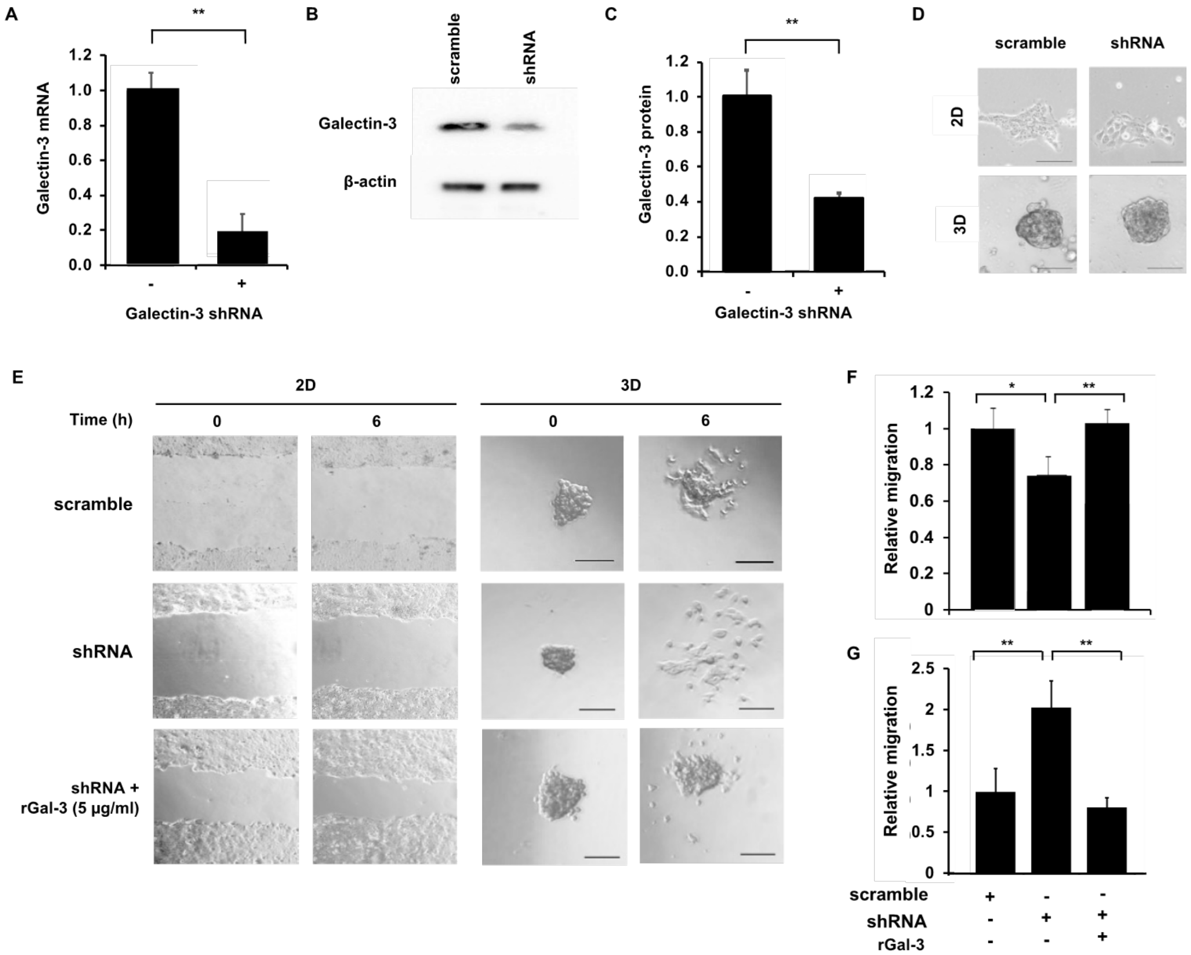

Figure S1 Effect of galectin-3 on 2D and 3D RBE cell migration. (A) The relative galectin-3 mRNA level of RBE knockdown cells compared to the control. (B) Representative galectin-3 immunoblots of RBE cells subjected to *gal-3* and scrambled shRNA. β-actin was used as the loading control. (C) The relative galectin-3 protein expression of RBE knockdown cells compared to the control. (D) The morphology of galectin-3 knockdown cells in 2D and 3D cultures. (E) Control and galectin-3 knockdown RBE cells were grown as monolayer or tumor spheroids for 4 days. rGal-3 was treated during the migration assay. The migration area of RBE monolayer and tumor spheroids were collected at 0 h and 6 h. The scale bars represent 100 µm. (F, G) Bars represent the relative migration of galectin-3 knockdown cells with and without the treatment of rGal-3 in (F) 2D and (G) 3D conditions compared to the control cells. The data represent means and ± standard error. * $p < 0.05$, ** $p < 0.01$.
